# Rapid differentiation of soil and root microbiomes in response to plant composition and biodiversity in the field

**DOI:** 10.1038/s43705-023-00237-5

**Published:** 2023-04-19

**Authors:** Haley M. Burrill, Guangzhou Wang, James D. Bever

**Affiliations:** 1 The University of Kansas, Lawrence, KS USA; 2 China Agricultural University, Beijing, China

**Keywords:** Microbiome, Fungal ecology, Microbial ecology

## Abstract

Research suggests that microbiomes play a major role in structuring plant communities and influencing ecosystem processes, however, the relative roles and strength of change of microbial components have not been identified. We measured the response of fungal, arbuscular mycorrhizal fungal (AMF), bacteria, and oomycete composition 4 months after planting of field plots that varied in plant composition and diversity. Plots were planted using 18 prairie plant species from three plant families (Poaceae, Fabaceae, and Asteraceae) in monoculture, 2, 3, or 6 species richness mixtures and either species within multiple families or one family. Soil cores were collected and homogenized per plot and DNA were extracted from soil and roots of each plot. We found that all microbial groups responded to the planting design, indicating rapid microbiome response to plant composition. Fungal pathogen communities were strongly affected by plant diversity. We identified OTUs from genera of putatively pathogenic fungi that increased with plant family, indicating likely pathogen specificity. Bacteria were strongly differentiated by plant family in roots but not soil. Fungal pathogen diversity increased with planted species richness, while oomycete diversity, as well as bacterial diversity in roots, decreased. AMF differentiation in roots was detected with individual plant species, but not plant family or richness. Fungal saprotroph composition differentiated between plant family composition in plots, providing evidence for decomposer home-field advantage. The observed patterns are consistent with rapid microbiome differentiation with plant composition, which could generate rapid feedbacks on plant growth in the field, thereby potentially influencing plant community structure, and influence ecosystem processes. These findings highlight the importance of native microbial inoculation in restoration.

## Introduction

Rapid biodiversity loss in the Anthropocene necessitates improved understanding of the ecological processes and factors that maintain biodiversity. While many gaps persist in our understanding of what drives biodiversity maintenance, accumulating evidence suggests that microbes can mediate plant species coexistence through feedbacks generated by host-specific differentiation of the microbiome [1]. Such plant-microbiome feedbacks have been shown to contribute to native plant diversity through negative feedbacks often resulting from accumulation of host-specific pathogens when plant diversity is low [2–6]. Alternatively, microbiome differentiation can generate alternative stable states through positive feedbacks, often through changes in density of microbial mutualists such as mycorrhizal fungi and rhizobia [6–8]. Additionally, litter decomposition rates have been found to differentiate among available plant species [9]. In this way, microbial phylotype composition and diversity can drive plant-soil feedback (PSF) dynamics, which can affect plant community composition and productivity. Critical questions remain in understanding the importance of microbiome feedbacks, including the patterns of differentiation amongst microbiome components, as well as how quickly they can change.

Plant microbiome feedback can be driven by multiple components of the plant and soil microbiome including pathogens, mutualists, and saprotrophs [10, 11]. The relative importance in generating feedback depends on microbiome components’ differential impacts on hosts, and the time it takes for differentiation on hosts [2, 12]. Most of our information on rates of differentiation of the plant and soil microbiome on host species comes from greenhouse assays. Greenhouse assays show, for example, that pathogen differentiation and pathogen-driven negative feedbacks can develop within a growing season [13, 14]. Similarly, greenhouse assays demonstrate that host-specific differentiation of AMF can occur over a growing season [4, 15]. In the field, pathogen, AMF, and saprophyte composition has been shown to vary with proximity to long-lived, mature plants such as trees [16–18]. Monitoring rates of differentiation in the field, however, requires manipulative experiments. The few such tests of differentiation of microbiome components have spanned multiple years and have targeted only a subset of the microbiome components [16, 18, 19]. More work directly comparing rates of host-species differentiation of microbiome components in the field would guide inference on their potential importance in generating rapid feedbacks.

The relative strength of host-specific differentiation of microbiome components are likely to be influenced by phylogenetic similarity of the host plants. Plant microbiome feedbacks have been shown to become increasingly negative with greater phylogenetic distance between hosts [3]. This is likely a result of co-evolution and specialization of pathogens with plant host defense mechanisms, as more closely related plant species are more likely to share functional traits, including defenses [5, 20, 21]. While AMF associate with hosts indiscriminately, phylogenetic signals have been detected in AMF impacts on host growth [22]. Thus, host phylogenetic patterns of AMF differentiation on hosts may be likely, but have not been tested to date. Finally, phylogenetic signals have also been found in saprotrophic community composition [18, 23]. The relative strength of phylogenetic structure on differentiation of each of these microbial community components has yet to be explored.

Microbiome composition can also be impacted by the diversity of plants within a community [24]. Specifically, microbiome differentiation is expected to diminish with the richness of local plant communities, potentially contributing to productivity responses to plant diversity [25–28]. Reduced densities of host specific pathogens with increasing plant diversity (i.e. dilution, [29]) is a likely mechanism driving productivity gains with increasing plant richness [1]. Mycorrhizal composition can also respond to plant diversity [30], potentially mediating benefits to neighbors and contributing to productivity gains with diversity [25, 27]. Changes in saprotroph composition with plant richness could potentially also contribute to greater productivity with high plant species richness. Decomposition rates may also be enhanced by increased plant diversity, reflecting changes in saprotroph composition [31–33]. While multiple microbiome components can contribute to changes in ecosystem function with plant biodiversity, their relative response to plant richness could determine the time-lags in productivity responses to plant diversity manipulation [25]. To date, we know little of the relative strength of change in these microbiome components with plant richness.

Plant driven changes in microbiome composition will likely depend upon the proximity of the microbes to roots. Host selection during microbial colonization of roots can act as a filter [34] and differences in root traits, including in root exudate production, likely drives differentiation of microbiome composition between plant species. Therefore, the composition of microbes in the roots may diverge more rapidly across plant species than microbes in soil. As a corollary, we might expect that pathogens and AMF, which interact with and colonize live root tissues, would differentiate more quickly than saprotrophic microbes, as they decompose dead and dying plant materials. We therefore expect stronger differentiation of pathogens and AMF in roots than in soil, and perhaps stronger differentiation of symbionts in the soil than saprotrophs. To date, few studies have measured the differentiation of microbiome components of plant roots compared to that of the surrounding soil [35].

In this study, we tested for soil microbiome differentiation across plant species of varying phylogenetic distance. We did this in the context of an experimental manipulation of plant biodiversity and composition in which positive productivity responses have been observed to plant species richness (Podzikowski, *pers comm*.). Specifically, we tested the response of soil and root microbiomes to manipulations of plant diversity, phylogenetic dispersion, and plant composition four months after planting *(see* Appendix Fig. 1*)*. Sequencing amplicons targeting bacteria, fungi, AMF and oomycetes from roots and soil separately across 120 plant community combinations allowed dissection of relative strength of microbiome differentiation across microbial functional and taxonomic groupings, and across soil and root compartments. Under the expectations that host-specific pathogens drive plant community composition, we expect fungal pathogens and oomycetes to differentiate more strongly than other, less host-specific microbial groups. We also expected root compartments to differentiate more strongly than soil compartments. Lastly, we expected that more strongly differentiating groups, i.e. groups with greater host specialization, would also show stronger responses to manipulations of plant diversity.

## Methods

### Study system

This study was conducted in the floristically diverse tallgrass prairie region of North America. Plots were established in June 2018 at the KU Field Station in Lawrence, KS, US (39.052462, −95.191656). Historically this land was tallgrass prairie, followed by cropland and pasture, today considered “post-agricultural” with predominantly cool-season nonnative grasses [36]. As part of the experiment setup, we tilled the resident soil and added soil made available because of road widening construction from an unplowed prairie remnant near Welda, KS, (38.179600, −95.265695) ~100 km south of the experiment site. This provided experimental plots with an initial microbial inocula of remnant prairie microbes.

### Experimental design

**Fig. 1:**
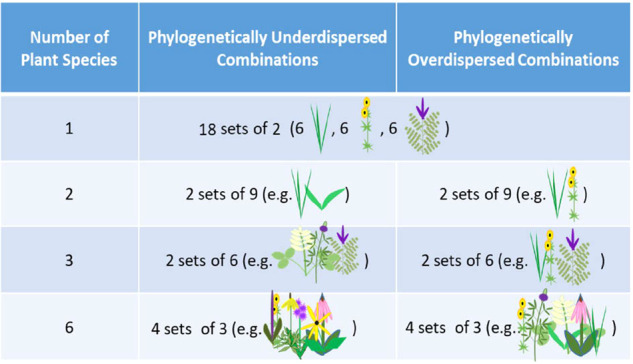
A factorial description of plot design. Number of plant species denotes the plant species richness treatments (monoculture, 2, 3, or 6 species). Phylogenetically under-dispersed combinations of plants are all within one plant family (e.g. 2 grasses, or 3 legumes, or 6 asters). Phylogenetically over-dispersed combinations of plants are from more than one plant family (e.g. 1 grass and 1 aster, or 1 species from each family, or 2 species from each family). Monocultures are inherently under-dispersed. The sets describe the combinations in which each of the 18 plant species are represented once (i.e. within sets species are randomly chosen without replacement from the pool of 18 plant species). There are 18 monocultures, two sets of 9 under-dispersed 2-plant plots, two sets of 9 over-dispersed 2-plant plots, two set of 6 under-dispersed 3-plant plots, two sets of 6 over-dispersed 3-plant plots, three sets of under-dispersed 6-plant plots, and three sets of over-dispersed 6-plant plots. This approach equally represents each plant species in each richness treatment by phylogenetic dispersion combination.

A total of 240 plots (1.5 m × 1.5 m) were designed to equally represent each of the 18 plant species (6 from each of the three plant families, Poaceae, Fabaceae, and Asteraceae) within each combination of plant species richness (1, 2, 3, and 6), phylogenetic dispersion (under or over), and precipitation (50 or 150% ambient). Plots varied in plant diversity, phylogenetic dispersion and composition across 72 monoculture plots, 72 with 2 species mixtures, 48 with 3 species mixtures, and 48 with 6 species (Fig. 1; Supplementary Appendix Fig. 1). These plots represent two replicates of the same 120 plant combinations, with half set up to receive 150% water treatment (150% of annual precipitation), while the other 120 replicated plots would receive 50% water (50% of annual precipitation). However, this water treatment began after samples for this analysis were collected and therefore precipitation effects will not be considered in these analyses. We describe the full design here, so that data collected in subsequent years can build off this initial analysis. Soil samples collected from these replicate future precipitation treatments were pooled prior to analysis, for a total of 120 pooled samples: 36 monocultures, 36 two-species, 24 three-species, and 24 six-species. Two-species plots either contained two plant families (Poaceae and Fabaceae, Poaceae and Asteraceae, or Fabaceae and Asteraceae) to represent over-dispersion; three- and six-species plots either contained all three families (over-dispersion) or species all within one plant family (under-dispersion) (Fig. 1). We analyzed phylogenetic dispersion by creating 4 categories: multi-family, under-dispersed Poaceae, under-dispersed Fabaceae, and under-dispersed Asteraceae.

### Experiment details

Prairie seedlings from the three most common plant families (Poaceae, Fabaceae, Asteraceae) were planted in May 2018. A total of 18 species were used (Supplementary Appendix Table 1). Seeds were purchased from producers located near eastern Kansas: Hamilton Native Outpost, Stock Seed, Missouri Wildflowers, and Prairie Moon. Each plot was seeded with each plant species being equally represented by pre-weighing 100 seeds per species and evenly dividing the final mix of species by weight, resulting in 1800 seeds per blend. Resident soil microbes were augmented with soil microbes in two ways: we tilled the resident soil to a depth of 15 cm, as well as added an average of 3.8 cm of soil from an unplowed native prairie soil from Welda, KS to each plant plug.

In addition, 18 seedlings previously inoculated with native Welda soil were planted into each plot. Seeds were sowed into flats with autoclaved sterile potting soil and placed in cold-moist stratification for 4 weeks prior to germination. When large enough, the seedlings were transplanted into Stuewe and Sons groove tubes (GT51D) with 98 mL of Welda soil and grown in a greenhouse for 5 weeks prior to being planted into a hexagonal array within each plot. Plugs were planted following the experimental design May 2018 and each plot was weeded according to the treatment during the summer months. Prior to sampling, only one round of weeding was accomplished, removing all forbs but leaving any grasses below 3 inches.

### Soil collection & DNA extraction

In September 2018, approximately 4 months after planting, soil was collected from each of the 240 plots. A total of two 20 cm soil cores were taken from each plot, added to a sample bag, and then paired plots of matched plant composition were pooled between the future rainfall treatments (i.e., 1,21; 2,22; 3,23; etc.; Supplementary Appendix Fig. 1). Coring devices were rinsed of dirt in a water bucket, then sterilized in 10% bleach bucket and wiped with 80% ethanol between plots. Immediately following soil collection, samples were kept on ice, then later transferred to a -20 °C freezer within 5 h [37]. Homogenized samples were thawed to sieve out roots and, following the Qiagen DNeasy PowerSoil kit, 0.25 g of the remaining soil was weighed for DNA extraction, as well as 0.25 g of roots separate from soil.

### Microbial community library preparation

Bacterial, fungal, oomycete, and AM fungal communities were sequenced from both soil and root DNA. For all communities, we used a two-step PCR process: the first PCR reactions use community-specific primers to amplify those regions of rDNA, followed by a clean-up using AMPure XP beads (Beckman Coulter, Brea, CA, USA), then a second PCR to bind unique barcode combinations using Nextera XT Index Kit v2 (Illumina, San Diego, CA, USA), and finally another AMPure XP bead clean-up. Following each PCR, PCR product were checked on 1.5% (w/v) agarose gel to estimate the quality of PCR products. PCR products concentration was measured by Invitrogen Qubit 3.0 Fluorometer (Thermo Fisher Scientific, Waltham, MA, USA). Adaptor ligation and sequencing was performed by Illumina MiSeq v3 PE300 Next-Gen Sequencer in Genome Sequencing Core (GSC) at the University of Kansas. Raw sequencing data are available at NCBI Sequence Read Archive, BIOPROJECT #PRJNA863284.

For fungi, AMF, and bacteria, the first PCR used a mixture of 1 μl sample DNA, 10.5 μl ddH_2_O, 0.5 μl each of forward and reverse primer and 12.5 μl of Master Mix Phusion (Thermo Fisher Scientific, Waltham, MA, USA), for a total PCR volume of 25 μl. For these communities the second barcoding PCR used 5 μl cleaned up sample DNA from the first PCR, 10.5 μl ddH_2_O, 2.5 μl each of forward and reverse barcode primers, and 25 μl of Master Mix Phusion, for a total volume of 45 μl.

The primers used for fungi target the internal transcribed spacer (ITS) regions forward fITS7 (5’-GTGAGTCATCGAATCTTTG-3’) and reverse ITS4 (5’-TCCTCCGCTTATTGATATGC-3’) [38]. The first PCR cycle for fungi began at 94 °C for 5 min, followed by 35× (94 °C for 30 s, 57 °C for 30 s, 72 °C for 30 s), 72 °C for 7 min, ending on 4 °C until retrieved from the thermocycler. The barcode PCR cycle began at 98 °C for 30 s, followed by 10× (98 °C for 10 s, 55 °C for 30 s, 72 °C for 30 s), 72 °C for 5 min, ending on 4 °C until retrieved from the thermocycler.

We used forward fLROR (5’-ACCCGCTGAACTTAAGC-3’) and reverse FLR2 (5’- TCGTTTAAAGCCATTACGTC-3’) primers to target the large subunit (LSU) region of AM fungi [39, 40]. The first PCR cycle for AMF began at 94 °C for 5 min, followed by 35x (94 °C for 30 s, 48 °C for 30 s, 72 °C for 30 s), 72 °C for 10 min, ending on 4 °C until retrieved from the thermocycler. The barcode PCR cycle was the same as for fungi.

For bacteria, we used primers that target the V4 region of 16S small subunit (SSU) of ribosomal RNA, forward 515F (5’-GTGYCAGCMGCCGCGGTAA-3’) and reverse 806R (5’-GGACTACNVGGGTWTCTAAT-3’) [41]. The first PCR cycle and the barcode PCR cycles were the same for bacteria as for fungi.

For oomycetes, we targeted ITS using forward ITS300 (5’-AGTATGYYTGTATCAGTGTC-3’) and reverse ITS4 (5’-TCCTCCGCTTATTGATATGC-3’). The first PCR used a mixture of 1 μl sample DNA, 17 μl ddH_2_O, 1 μl each of forward and reverse primer and 5 μl of HOT FIREPol (Solis Biodyne, Tartu, Estonia), for a total volume of 25 μl. The first PCR cycle for oomycetes began at 95 °C for 15 min, followed by 35x (95 °C for 30 s, 55 °C for 30 s, 72 °C for 1 min), 72 °C for 10 min, ending on 4 °C until retrieved from the thermocycler. The second barcoding PCR used 1 μl cleaned up sample DNA from the first PCR, 18 μl ddH_2_O, 0.5 μl each of forward and reverse barcode primers, and 5 μl of HOT FIREPol, for a total volume of 45 μl. The oomycete barcode PCR cycle began at 95 °C for 15 min, followed by 35× (95 °C for 30 s, 55 °C for 30 s, 72 °C for 1 min), 72 °C for 10 min, ending on 4 °C until retrieved from the thermocycler.

### Bioinformatics

After sequencing, the primary analysis of raw FASTQ data was processed with the QIIME2 pipeline [42]. After sequences were demultiplexed and primers removed, they were quality filtered, trimmed, de-noised, and merged using DADA2 [43]. Chimeric sequences were identified and removed via the consensus method in *dada2*. The OTUs that only appeared 5 times or fewer across all samples were discarded to preclude inclusion of sequences from potential contamination or sequencing errors. Taxonomy was assigned to all ribosomal sequence variants in QIIME2 using a feature classifier trained with the SILVA 99% OTU database for bacteria [44] and the UNITE 99% database for fungi (Version 18.11.2018). This resulted in 2022 bacteria OTUs in the roots and 1261 in the soil. We used the FUNGuild database to identify putative pathogens, those labeled trophic mode “Pathotroph” and guild “plant pathogen” from the fungal sequences [45]. For saprotrophs, we filtered all trophic modes that include “Saprotroph” but do not include “Pathotroph” and removed all guilds that did not include saprotrophs of plant materials. For both putative pathogens and saprobes, we removed confidence rankings “possible,” as per the authors’ recommendations [45]. For soil, 1904 out of 7272 OTUs were matched to a guild, 254 of which were putative pathogens and 727 were putative saprotrophs. For roots, 964 of 3650 OTUs were matched to a guild, 133 of which were putative pathogens and 346 were saprotrophs. For AMF LSU amplicons, we excluded non-AMF sequences by building a phylogenetic tree using the curated database base of AMF [46] using *Mortierella elongata* sequences as the outgroup [39, 40]. This resulted in 2395 AMF OTUs in the roots and 8230 in soil. For oomycetes, we checked the identity of resulting OTUs either against a database containing all NCBI oomycote ITS2 sequence results using the Basic Local Alignment Search Tool, BLAST v. 2.6.0 [47], using default parameters, or by placing OTUs in the oomycete clade, as oomycota are thought to have arisen from a common ancestor forming a conserved clade [48]. This resulted in 141 oomycete OTUs in the roots and 460 in soil. We make the generalization that terrestrial oomycetes are primarily parasites of vascular plants [49, 50] and those found in our plots are likely to function as plant pathogens.

### Statistical analysis

**Fig. 2:**
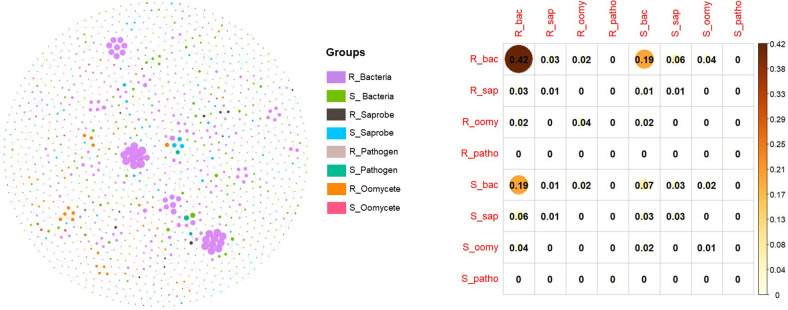
Networks analysis revealing the co-occurrence patterns between microbial groups. The nodes were colored according to group labels. A connection represents a strong (Spearman’s correlation coefficient *r* > 0.8) and significant (*p* < 0.001) correlation. The size of each node is proportional to the number of connections. “R”, “S” represent root and soil samples, respectively.

To investigate co-occurrence patterns among different groups of microbial community in roots and soil, a correlation matrix was constructed by calculating all possible pairwise Spearman’s rank correlations among the OTUs. Network analysis was performed in R environment (version 4.0.2) using *vegan*, *igraph* and *hmisc* packages and the visualization was conducted on the interactive platform of Gephi 0.9.1. A correlation between two items was considered statistically robust if the Spearman’s correlation coefficient (ρ) was >0.7 and the *P* value was <0.001 [51]. To reduce the chances of obtaining false-positive results, the *P* values were adjusted with a multiple testing correction using the Benjamini-Hochberg method [52]. The number of connections between each pair of OTUs were counted, and the proportion of interactions within and between groups were calculated and visualized using *corrplot* package (Fig. 2).

#### Microbial diversity

**Fig. 3:**
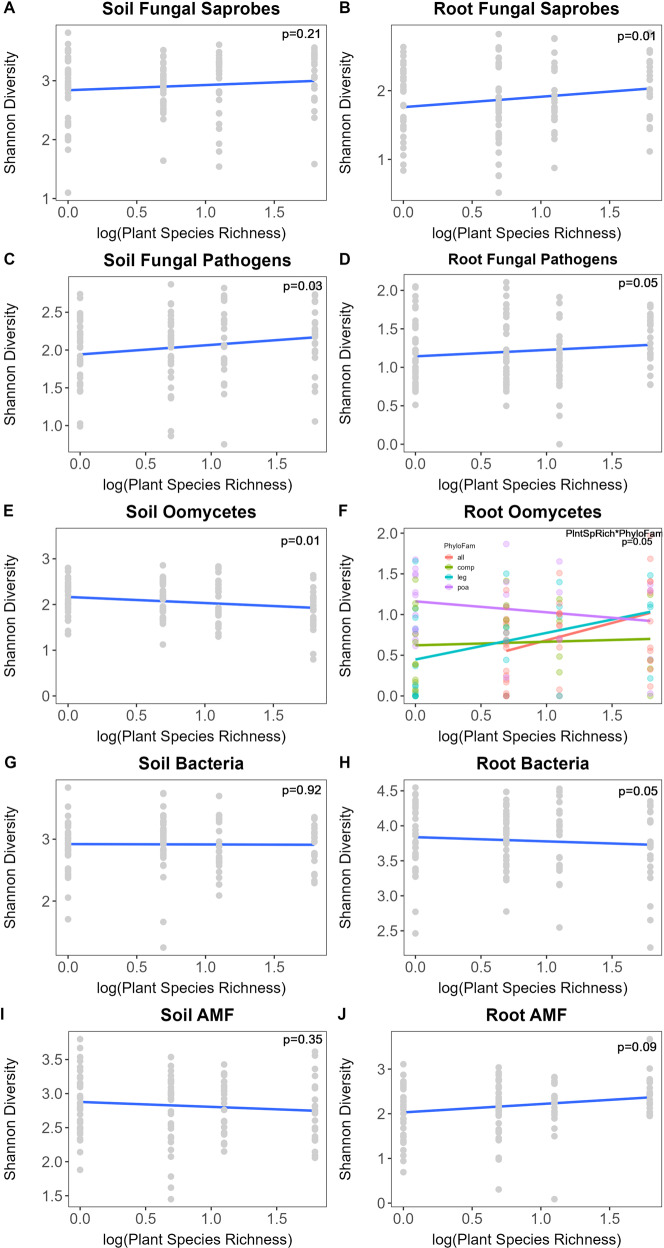
Microbial diversity response to plant species richness treatment. Regression of (**a**) soil (*p* > 0.1) and (**b**) root fungal saprotroph diversity (*p* = 0.01), (**c**) soil (*p* = 0.03) and (**d**) root (*p* = 0.05) pathogenic fungal diversity, (**e**) soil (*p* = 0.01) and (**f**) root (*p* = 0.05, interaction with family treatment) oomycete diversity, (**g**) soil (*p* > 0.1) and (**h**) root (*p* = 0.05) bacteria diversity, (**i**) soil (*p* > 0.1) and (**j**) root (*p* = 0.09) AMF diversity in response to the log-transformed planted species richness treatment. Diversity responses were adjusted when plant family composition or block had significant responses. *P*-values reported are from GLM of microbial diversity response to the model, as shown in detail in Table 1.

To assess planting effects on microbial diversity, we ran general linear models for each community diversity (H’) with the experimental design model: using block, planted species richness, phylogenetic dispersion, as well as planted proportions of each of the 18 plant species as fixed effects, with one interaction term for plant species richness and phylogenetic dispersion. Using the *vegan* package, we calculated Shannon-Wiener diversity [53]. We used the Shannon-wiener index for relative abundance of OTUs, as it accounts for both richness and evenness which thus allows for detection of more rare OTUs; see Supplementary Appendix Fig. 2 for rarefaction curves created using the *adiv* package in R [54]. A regression Fig. was created in ggplot2 to visualize significant responses of microbial groups to plant species richness treatments (Fig. 3).

#### Microbial composition

First, OTU tables were centered log-ratio transformed, using the *transform* function in r [55]. Then, we created a Bray-Curtis dissimilarity matrix using the diversity function in the *vegan* package [53]. Using the dissimilarity matrices of non-pathogenic fungi, putative fungal pathogens, bacteria, oomycetes, and AMF, in both soil and roots, we performed PERMANOVA tests to assess the variance explained by block (for spatial non-independence), planted species richness (“PlntDiv”), phylogenetic dispersion (“PhyloFam”), the planted proportions of each of the 18 species per plot, as well as an interaction term for plant species richness and phylogenetic dispersion (see Supplementary Appendix Table 1*)*. We ran analyses for beta dispersion (spread of microbial community composition within samples) using the *vegan betadisper* function for all significant responses to plant species richness and plant family composition.

In order to understand what may be driving the significant effects seen in the PERMANOVA analyses of microbial communities, we ran Principle Coordinate Analyses using the *pco* function in the *ecodist* package in R (version 4.0.3) [56]. We ran the full model with each PC axis as a response variable for each community to indicate whether each axis may be driving significant differences in the multi-categorical phylogenetic dispersion.

To assess the response of groups to plant family composition, we calculated the relative abundance of soil fungal pathogens and root fungal pathogens within taxonomic groups using usearch10 [57]. This uses the taxonomic databases of the groups to assign OTUs to phyla, family, genus, and some to species. Then it calculates relative abundance of each taxonomic group, based on the raw OTU reads from the bioinformatics pipeline output. We ran the model in a general linear model to detect the response of relative abundances of putative pathogen OTUs within each detected fungal genera to plant family composition and we report genera with significantly different relative abundances.

## Results

### Roots vs. soil

In our co-occurrence network analyses, the only group that had significant correlation between root and soil community composition was bacteria (Fig. 2). We therefore proceed to report results for roots and soil communities separately.

### Microbial diversity

Fungal pathogen diversity in the soil had a positive response to plant species richness (Table 1, Fig. 3C; *F*_1,93_ = 4.89, *p* = 0.03), but oomycete diversity in the soil decreased with plant species richness (Table 1, Fig. 3E; *F*_1,93_ = 6.22, *p* = 0.01). Neither bacteria nor AMF diversity in the soil responded significantly to plant species richness.

**Table 1:** Soil microbial community diversity (H’) response to experimental design model.

Soil		Fungal saprobes		Fungal pathogens		Oomycetes		Bacteria		AMF	
	Df	*F* value	Pr(>*F*)	*F* value	Pr(>*F*)	*F* value	Pr(>*F*)	*F* value	Pr(>*F*)	*F* value	Pr(>*F*)
Block	1	0.90206	0.34	2.04902	0.16	3.75354	0.056	0.77341	0.38	36.2602	4.1E−08
PhyloFam	3	0.64488	0.59	0.06835	0.98	0.24882	0.86	0.37813	0.77	1.27734	0.29
**PlntDiv**	1	1.60715	0.21	4.88864	**0.029**	6.21715	**0.014**	0.00797	0.93	0.86611	0.35
SCHSCO	1	0.64644	0.42	0.22783	0.63	2.29249	0.13	0.25116	0.62	0.00283	0.96
ANDGER	1	5.27578	**0.02**	0.01058	0.92	0.0009	0.98	2.14351	0.15	2.51044	0.12
KOEMAC	1	0.24281	0.62	2.1734	0.14	0.06516	0.80	0.17531	0.67	3.73366	**0.057**
ELYCAN	1	7.26972	**0.008**	2.17314	0.14	0.17282	0.68	0.16693	0.68	7.5E−05	0.99
BOUGRA	1	0.00679	0.93	0.05478	0.82	0.03728	0.85	0.86503	0.35	0.31383	0.58
PANVIR	1	0.66727	0.42	0.30194	0.58	0.01928	0.89	0.18795	0.67	0.35363	0.55
AMOCAN	1	1.05058	0.31	1.32978	0.25	0.6418	0.43	0.32182	0.57	0.00063	0.98
DALCAN	1	6.8E−06	0.99	0.28936	0.59	3.99125	**0.049**	2.62044	0.11	0.07392	0.79
DALPUR	1	0.4242	0.52	0.12544	0.72	0.00398	0.95	2.50553	0.12	0.34743	0.56
DESILL	1	0.10366	0.75	0.70462	0.40	0.34597	0.56	0.00019	0.99	2.67088	0.11
DESCAN	1	0.95336	0.33	0.52304	0.47	0.00021	0.99	3.25769	0.074	1.11198	0.29
CHAFAS	1	0.63978	0.43	1.01918	0.32	3.43685	**0.067**	1.11498	0.29	2.51816	0.12
LIAPYC	1	4.49713	**0.037**	0.85479	0.36	1.84872	0.18	0.06267	0.80	3.58524	**0.062**
CORTIN	1	0.21805	0.64	4.58312	**0.035**	0.4489	0.50	0.32741	0.57	2.02827	0.16
ECHPAL	1	2.28555	0.13	0.02942	0.86	2.40844	0.12	1.61026	0.21	0.00265	0.96
EUPALT	1	0.12049	0.73	0.01108	0.92	0.01853	0.89	0.18979	0.66	0.06732	0.80
SILINT	1	3.25838	**0.074**	0.90128	0.34	0.41998	0.52	0.02364	0.88	0.36919	0.55
HELMOL	1	0.01351	0.91	0.53675	0.47	0.53217	0.47	0.74395	0.39	0.25435	0.62
PhyloFam:PlntDiv	3	1.90035	0.13	1.79088	0.15	0.05285	0.98	0.45262	0.72	0.75247	0.52
**Roots**		**Fungal saprobes**		**Fungal pathogens**		**Oomycetes**		**Bacteria**		**AMF**	
	**Df**	***F*** **value**	**Pr(>*F*)**	***F*** **value**	**Pr(>*F*)**	***F*** **value**	**Pr(>*F*)**	***F*** **value**	**Pr(>*F*)**	***F*** **value**	**Pr(>*F*)**
Block	1	1.5E−05	0.99	0.00411	0.95	0.57233	0.45	6.84882	**0.010**	0.02712	0.87
PhyloFam	3	0.1448	0.93	0.70493	0.55	4.55984	**0.0051**	2.71979	**0.050**	2.26228	**0.087**
**PlntDiv**	1	6.77016	**0.011**	3.88328	**0.052**	3.41045	**0.068**	3.93536	**0.050**	3.002	**0.087**
SCHSCO	1	0.03023	0.86	0.00107	0.97	1.23257	0.27	0.06219	0.80	2.3779	0.13
ANDGER	1	0.07522	0.78	0.98278	0.32	0.46704	0.50	1.03043	0.31	3.50869	**0.064**
KOEMAC	1	0.48424	0.49	0.06106	0.81	0.02149	0.88	0.10873	0.74	0.43347	0.51
ELYCAN	1	0.42569	0.52	0.60896	0.44	0.8242	0.37	1.62471	0.21	0.98493	0.32
BOUGRA	1	0.03591	0.85	0.54603	0.46	0.25143	0.62	0.72409	0.40	0.68515	0.41
PANVIR	1	0.04607	0.83	2.49732	0.12	0.93479	0.34	0.00023	0.99	0.95705	0.33
AMOCAN	1	0.10767	0.74	0.4635	0.50	0.9011	0.35	3.27726	**0.073**	4.93726	**0.029**
DALCAN	1	3.63083	**0.060**	0.6616	0.42	3.29542	**0.073**	0.0577	0.81	0.00232	0.96
DALPUR	1	4.94435	**0.029**	0.00074	0.98	0.27084	0.60	1.8177	0.18	0.00073	0.98
DESILL	1	0.03831	0.85	0.09956	0.75	0.93316	0.34	4.45755	**0.037**	5.08429	**0.027**
DESCAN	1	0.20625	0.65	0.40431	0.53	1.14546	0.29	0.50886	0.48	0.62382	0.43
CHAFAS	1	0.0872	0.77	0.67301	0.41	1.04541	0.31	0.74925	0.39	1.73202	0.19
LIAPYC	1	0.04845	0.83	0.90113	0.34	2.09055	0.15	1.14682	0.29	0.55573	0.46
CORTIN	1	0.79847	0.37	0.07857	0.78	4.65008	**0.034**	0.0934	0.76	0.01955	0.89
ECHPAL	1	0.00248	0.96	0.1288	0.72	0.09223	0.76	1.12969	0.29	2.9961	**0.087**
EUPALT	1	0.21802	0.64	1.67995	0.20	1.07625	0.30	1.66151	0.21	1.12731	0.29
SILINT	1	5.22179	**0.025**	0.2039	0.65	0.30121	0.58	0.02523	0.87	0.23837	0.63
HELMOL	1	0.08907	0.77	0.21556	0.64	3.08988	**0.082**	3.4421	**0.067**	0.42648	0.52
PhyloFam:PlntDiv	3	1.31543	0.27	1.13742	0.34	2.66017	**0.053**	0.44519	0.72	0.31464	0.81

“PlntDiv” refers to planted species richness treatment, “PhyloFam” refers to plant family categories – mixtures, under-dispersed Asteraceae, Poaceae, or Fabaceae – followed by presence of each plant species, as well as the interaction of plant family with species richness.

Significant (<0.05) and marginal (<0.1) *p*-values are shown in bold.

In roots, both fungal pathogen (Table 1, Fig. 3D; *F*_1,92_ = 3.88, *p* = 0.05) and fungal saprobe (Table 1, Fig. 3B; *F*_1,92_ = 6.77, *p* = 0.01) diversity increased with plant species richness. Root oomycete diversity (Table 1, Fig. 3F; *F*_1,92_ = 3.41, *p* = 0.07) and AMF diversity (Table 1, Fig. 3J; *F*_1,92_ = 3.00, *p* = 0.09) had marginally significant increase with plant species richness. However, root bacteria diversity decreased with plant species richness (Table 1, Fig. 3H; *F*_1,92_ = 3.94, *p* = 0.05).

### Microbial community response

The composition of all microbial communities responded to the planting design; however, only fungal pathogens and root bacteria responded significantly to plant family composition and/or diversity (Table 2). Soil fungal pathogen community composition was significantly differentiated among plant species richness (Table 2, *p* = 0.03), and planted proportion of Fabaceae species *C. fasciculata* (Table 2, *p* = 0.01). We also highlight that there were marginal responses to planted proportions of Poaceae species *S. scoparium* (Table 2, *p* = 0.05) and Asteraceae species *C. tinctoria* (Table 2, *p* = 0.06). Soil fungal saprobes had a significant response to the planted proportion of Fabaceae species *C. fasciculata* (Table 2, *p* = 0.05), as well as marginally significant response to other Fabaceae species *A. canescens* (Table 2, *p* = 0.09), *D. canadense* (Table 2, *p* = 0.09), and Poaceae species *E. canadensis* (Table 2, *p* = 0.07). Additionally, soil oomycete composition was significantly different in response to planted proportions of *E. pallida* (Table 2, *p* = 0.05), and root bacteria composition had a significant response to the interaction of plant family composition and plant species richness treatments (Table 2, *p* = 0.02).

**Table 2:** PERMANOVA table for soil community composition (Bray−Curtis dissimilarity) response to experimental design.

Soil		Fungal saprotrophs		Fungal pathogens		Oomycetes		Bacteria		AMF	
	DF	R2	Pr(>*F*)	R2	Pr(>*F*)	R2	Pr(>*F*)	R2	Pr(>*F*)	R2	Pr(>*F*)
Block	1	0.025	0.001	0.017	0.006	0.038	0.001	0.010	0.162	0.027	0.001
PhyloFam	3	0.025	0.4	0.028	0.187	0.025	0.43	0.024	0.677	0.026	0.668
PlntDiv	1	0.008	0.401	0.014	**0.031**	0.005	0.962	0.007	0.808	0.008	0.695
SCHSCO	1	0.010	0.165	0.012	**0.082**	0.010	0.237	0.009	0.255	0.008	0.862
ANDGER	1	0.009	0.236	0.008	0.5	0.007	0.744	0.008	0.591	0.007	0.977
KOEMAC	1	0.008	0.502	0.008	0.525	0.005	0.953	0.009	0.171	0.008	0.819
ELYCAN	1	0.011	**0.074**	0.008	0.432	0.008	0.413	0.010	0.108	0.009	0.33
BOUGRA	1	0.006	0.879	0.005	0.93	0.006	0.873	0.008	0.652	0.009	0.508
PANVIR	1	0.009	0.327	0.009	0.307	0.010	0.18	0.009	0.153	0.009	0.393
AMOCAN	1	0.011	**0.086**	0.006	0.744	0.007	0.577	0.010	0.122	0.009	0.279
DALCAN	1	0.011	**0.088**	0.008	0.485	0.009	0.388	0.009	0.362	0.008	0.715
DALPUR	1	0.008	0.553	0.004	0.99	0.008	0.411	0.008	0.633	0.009	0.344
DESILL	1	0.006	0.918	0.011	0.171	0.006	0.805	0.008	0.544	0.008	0.761
DESCAN	1	0.007	0.784	0.008	0.518	0.006	0.777	0.009	0.318	0.009	0.508
CHAFAS	1	0.011	**0.05**	0.015	**0.01**	0.009	0.281	0.008	0.494	0.008	0.584
LIAPYC	1	0.010	0.127	0.010	0.18	0.008	0.409	0.009	0.397	0.009	0.562
CORTIN	1	0.010	0.172	0.013	**0.06**	0.008	0.527	0.008	0.472	0.010	0.208
ECHPAL	1	0.008	0.487	0.006	0.841	0.013	**0.05**	0.010	0.097	0.008	0.826
EUPALT	1	0.009	0.307	0.005	0.892	0.009	0.276	0.007	0.754	0.008	0.721
SILINT	1	0.007	0.799	0.007	0.691	0.006	0.839	0.006	0.988	0.009	0.313
HELMOL	1	0.010	0.187	0.004	0.898	0.005	0.919	0.008	0.736	0.007	0.943
PhyloFam:PlntDiv	3	0.027	0.187	0.028	0.233	0.019	0.921	0.030	**0.016**	0.024	0.919
Residual	93	0.756		0.766		0.771		0.776		0.762	
Total	119	1.000		1.000		1.000		1.000		1.000	
**Roots**		**Fungal saprotrophs**		**Fungal pathogens**		**Oomycetes**		**Bacteria**		**AMF**	
	**DF**	**R2**	**Pr(>*F*)**	**R2**	**Pr(>*F*)**	**R2**	**Pr(>*F*)**	**R2**	**Pr(>*F*)**	**R2**	**Pr(>*F*)**
Block	1	0.011	0.101	0.013	0.085	0.009	0.35	0.010	0.065	0.013	0.001
PhyloFam	3	0.025	0.441	0.026	0.405	0.034	**0.061**	0.103	**0.001**	0.026	0.807
PlntDiv	1	0.011	**0.085**	0.009	0.334	0.011	0.16	0.010	**0.086**	0.009	0.209
SCHSCO	1	0.009	0.389	0.008	0.537	0.011	0.136	0.008	0.26	0.010	**0.02**
ANDGER	1	0.009	0.256	0.012	0.158	0.008	0.534	0.008	0.235	0.008	0.809
KOEMAC	1	0.008	0.504	0.004	0.95	0.008	0.452	0.008	0.314	0.007	0.991
ELYCAN	1	0.010	0.211	0.005	0.905	0.010	0.255	0.008	0.242	0.009	0.239
BOUGRA	1	0.008	0.556	0.009	0.324	0.006	0.885	0.008	0.301	0.009	0.385
PANVIR	1	0.011	0.111	0.008	0.485	0.009	0.346	0.014	**0.005**	0.009	0.445
AMOCAN	1	0.008	0.575	0.004	0.924	0.010	0.267	0.017	**0.002**	0.010	**0.028**
DALCAN	1	0.012	**0.033**	0.006	0.726	0.010	0.243	0.011	**0.034**	0.009	0.598
DALPUR	1	0.008	0.492	0.005	0.918	0.008	0.554	0.018	**0.001**	0.010	0.104
DESILL	1	0.007	0.837	0.010	0.238	0.008	0.56	0.008	0.266	0.010	**0.05**
DESCAN	1	0.007	0.683	0.009	0.398	0.008	0.498	0.005	0.849	0.009	0.747
CHAFAS	1	0.007	0.794	0.006	0.751	0.007	0.742	0.011	**0.041**	0.008	0.889
LIAPYC	1	0.006	0.919	0.007	0.654	0.007	0.635	0.013	**0.008**	0.008	0.827
CORTIN	1	0.008	0.585	0.013	0.091	0.009	0.311	0.013	**0.006**	0.009	0.366
ECHPAL	1	0.009	0.415	0.017	**0.02**	0.004	0.986	0.007	0.402	0.009	0.221
EUPALT	1	0.008	0.464	0.008	0.488	0.008	0.451	0.010	**0.059**	0.009	0.472
SILINT	1	0.008	0.46	0.009	0.358	0.010	0.262	0.015	**0.005**	0.009	0.274
HELMOL	1	0.011	**0.048**	0.008	0.582	0.008	0.688	0.011	**0.025**	0.009	0.245
PhyloFam:PlntDiv	3	0.032	**0.028**	0.031	0.159	0.032	0.103	0.027	**0.065**	0.026	0.585
Residual	93	0.767		0.773		0.763		0.657		0.763	
Total	119	1.000		1.000		1.000		1.000		1.000	

Block is based on the spatial orientation on plots (see Supplementary Appendix Fig. 1). PhyloFam is the phylogenetically under-dispersed (either Poaceae, Fabaceae, or Asteraceae), or phylogenetically over-dispersed (2 or 3 plant families present). PlntDiv is the plant species richness treatment (1, 2, 3, or 6).

Significant (<0.05) and marginal (<0.1) *p*-values are shown in bold.

Root fungal saprobe composition differentiated by the planted proportion *of H. mollis* (Table 2, *p* = 0.05*), D. canadense* (Table 2, *p* = 0.03), and the interaction of plant family composition and plant species richness treatments (Table 2, *p* = 0.03); we also note a marginally significant response to plant species richness (Table 2, *p* = 0.09). Root fungal pathogen composition differentiated significantly among planted proportion of Asteraceae species *C. tinctoria* (Table 2, *p* = 0.02). Root oomycetes had a marginal response to plant family composition (Table 2, *p* = 0.06). Root bacterial community composition differentiated by plant family composition (Table 2, *p* = 0.001), planted species in Fabaceae (*p* = 0.002, *A. canescens*; *p* = 0.03, *D. canadense*; *p* = 0.001, *D. purpureum*), Poaceae (*p* = 0.005, *P. virgatum*), and Asteraceae (*p* = 0.008, *L. pycnostachya*; *p* = 0.006, *C. tinctoria*; *p* = 0.005, *S. integrifolium*; *p* = 0.03, *H. mollis*). In addition, root bacteria composition had a marginally significant response to plant species richness (Table 2, *p* = 0.09), the interaction of plant family composition and plant species richness (Table 2, *p* = 0.07), and planted proportion of Asteraceae species *E. altissimum* (Table 2, *p* = 0.06). Lastly, root AMF community composition differentiated with planted proportion of Poaceae species *S. scoparium* (Table 2, *p* = 0.02) and Fabaceae species *A. canescens* (Table 2, *p* = 0.03) and *D. illinoensis* (Table 2, *p* = 0.05).

Beta dispersion was not significant for soil fungal pathogen response to plant species richness (*F* = 0.294, *p* = 0.82) or root oomycete response to plant family composition (*F* = 1.064, *p* = 0.37). For root fungal saprobes, beta dispersion among plant species richness treatments was significantly different (*F* = 2.726, *p* = 0.04). Beta dispersion for root bacteria was significant among plant family composition treatments (*F* = 3.397, *p* = 0.02), but not for planted species richness (*F* = 1.921, *p* = 0.12).

### Drivers of community divergence

**Fig. 4:**
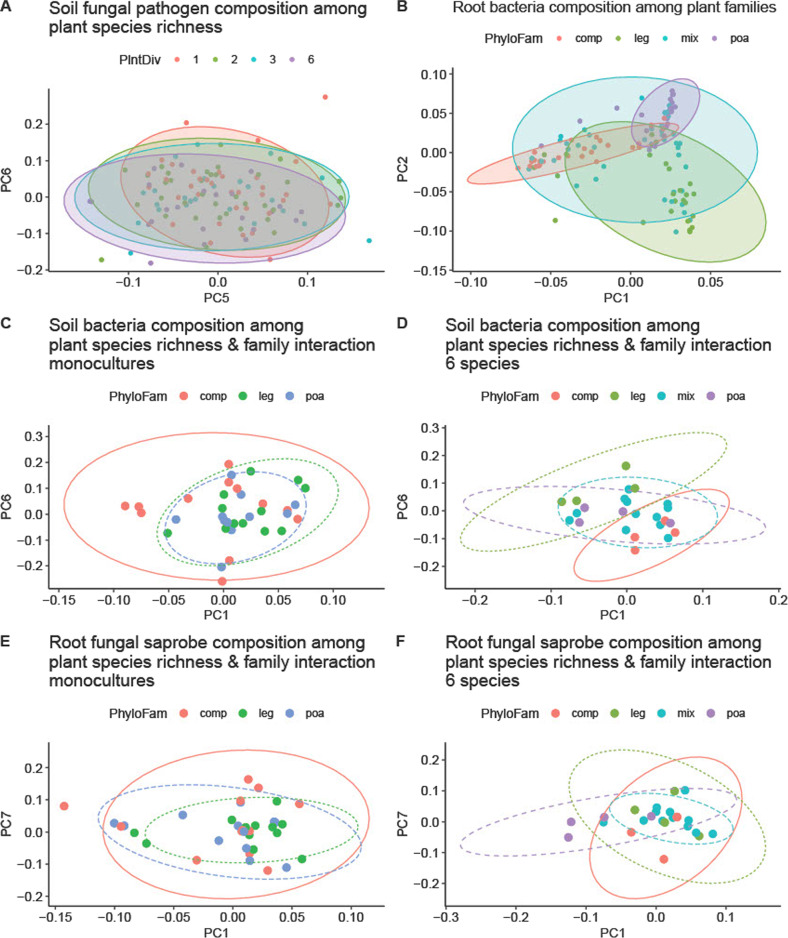
Microbial composition response to plant species richness and plant family composition treatments. PCA plots showing (**a**) soil fungal composition response to plant species richness (*p* = 0.03, Table 2), (**b**) root bacteria composition response to plant family (*p* = 0.001, Table 2), soil bacteria composition response to the interaction of plant species richness and plant family (*p* = 0.02, Table 2), monocultures depicted in **c** and 6-species treatment shown in **d**. 2-species and 3-species interaction plots found in Supplementary Appendix Fig. 3. Root fungal saprobe composition differentiation in response to the interaction of planted species richness and plant family (*p* = 0.03, Table 2) shown in monoculture (**e**) and 6-species (**f**), with 2- and 3-species plots found in Supplementary Appendix Fig. 3. *P*-values reported are from PERMANOVA of microbial bray-curtis dissimilarity response to the model, as shown in detail in Table 2.

To better understand the effects of plant family composition, we analyzed principal component coordinates for each microbial community that differed significantly with the plant family composition treatment (Fig. 4; Supplementary Appendix Table 2). Differentiation in soil fungal pathogens in response to plant family composition was detected in PC1 (*F*_3,93_ = 2.81, *p* = 0.04) and PC5 (*F*_3,93_ = 6.40, *p* = 0.001; Fig. 4A). In the roots, non-pathotrophic fungi responded to plant family composition in PC1 (*F*_3,85_ = 3.72, *p* = 0.01) PC2 (*F*_3,85_ = 2.11, *p* = 0.1; Fig. 4C), Oomycetes in PC4 (*F*_3,93_ = 2.33, *p* = 0.08) and PC6 (*F*_3,93_ = 3.54, *p* = 0.02; Fig. 4E), and bacteria in PC1 (*F*_3,93_ = 15.93, *p* = 1.9e−8) PC2 (*F*_3,93_ = 51.65, *p* = 2.2e−16; Fig. 4G).

**Fig. 5:**
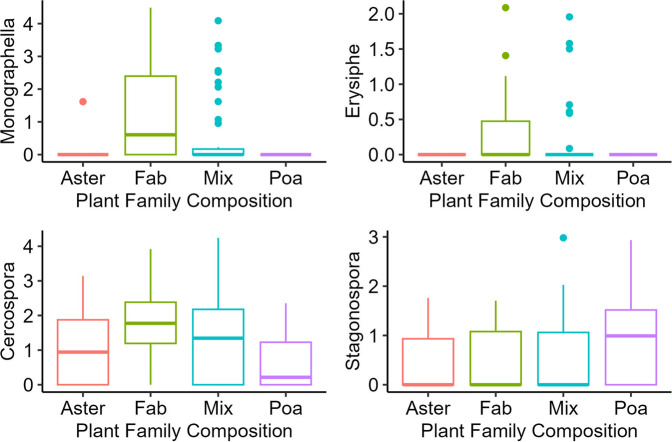
Differential relative abundance of fungal pathogen OTUs in response to plant family composition treatment. Fungal genera in soil with pathogens that respond significantly to the plant family treatment, “Mix” indicates over-dispersed treatments with more than one plant family, while “Aster”, “Fab”, and “Poa” indicate under-dispersed plots with only Asteraceae, Fabaceae, or Poaceae, respectively. Fungal genera on y axis are log-transformed relative abundance calculated from OTU counts. See Supplementary Appendix Table 3 for anova output.

Fungal genera in the soil that differentiated in relative abundance with plant family composition include putative pathogens in Monographella (*F*_3,93_ = 4.61, *p* = 0.005), Cercospora (*F*_3,93_ = 3.07, *p* = 0.03), and Erysiphe (*F*_3,93_ = 4.10, *p* = 0.009), all of which had higher abundance in plots with legumes only (Fig. 5). The relative abundance of putative pathogens within Stagonospora also differed significantly in response to plant family composition (*F*_3,93_ = 2.44, *p* = 0.07), with Poaceae diverging slightly (Fig. 5). In the roots, we found weak responses of putative pathogens within Papiliotrema (*F*_3,93_ = 2.69, *p* = 0.05) and Lophiostoma (*F*_3,93_ = 5.72, *p* = 0.001), which seem to be diverging by composites and grasses, respectively. See Supplementary Appendix Table 3 and Supplementary Appendix Fig. 4 for soil fungal family abundance.

## Discussion

We found rapid differentiation of the soil microbiome in response to plant community composition and diversity, as we observed pathogenic fungi, oomycetes, saprotrophic fungi, AMF, and bacteria in soil, roots or both compartments to be differentiate with planting design four months after planting (Table 2). These host-specific changes were largely independent of each other, as indicated by only weak and infrequent correlations being observed in the co-occurrence analyses (Fig. 2). We therefore analyzed individual microbial group responses to the experimental design separately. Of these microbial groups, fungal pathogens in soil and bacteria in roots responded most strongly in both total microbial community diversity (Fig. 3C, H) and composition (Fig. 4A, B) to manipulations of plant species richness and plant community composition, respectively. The differential strength of differentiation of these microbial groups suggests their relative potential importance in plant community dynamics.

### Relative strength of differentiation of microbial groups

Root bacteria and soil fungal pathogens differentiated most strongly with plant composition as they had the highest variation explained due to the model (Table 2), while AMF had the lowest rate of differentiation. Those detected differences were driven by plant family composition as well as individual plant species within the three planted families (Fig. 4B–F). Differentiation of microbiome composition can influence plant-plant interactions when these microbial components differentially impact host fitness [10]. Our results suggest that root colonizing bacteria and fungal pathogens are prime candidates for generating plant-soil feedback, while changes in AMF composition are less likely to be important in the short time frame. This is consistent with empirical tests of feedback [3]. While negative feedback through mycorrhizal differentiation within a growing season has been detected in the lab [15], meta-analyses of plant soil feedback show that feedbacks through non-AMF components like root and fungal pathogens are generally stronger and more negative [3].

Moreover, microbiome-driven plant soil feedback depends upon transmission of microbiomes from one plant to its neighbors. As root colonization comes from the pool of microbes in the soil, our detection of fungal pathogen differentiation on plant hosts in the soil is consistent with these fungal pathogens being available to serve as future colonists of neighboring plant roots. Oomycetes also had similar levels of differentiation in roots and soils. Together, this suggests that fungal and oomycete pathogens may be most likely to drive rapid negative plant-soil feedback, a result consistent with meta-analyses of plant soil feedback experiments [3] and with accumulating evidence of pathogens playing an important role in plant species coexistence [6, 58].

The divergence of fungal pathogens with plant family (Figs. 4A and 5) is consistent with pathogen specificity being structured by plant family, as has been shown previously [21]. The phylogenetic structure of pathogen specialization is also consistent with stronger negative feedbacks between phylogenetically distant plant pairs, as has been demonstrated in meta-analyses of feedback experiments [3]. Such feedback would generate stabilizing forces around phylogenetically over-dispersed plant communities. In the soil, fungal pathogens in the genera *Erypishe, Monographella*, and *Cercospora* were most abundant on Fabaceae (Fig. 5). While pathogens within *Cercospora* were relatively most abundant on Fabaceae, they were intermediate on Asteraceae, and the least abundant on Poaceae. In addition, pathogens within *Stagonospora* were most abundant on Poaceae, relative to the other plant families and mixture plots. These patterns of specialization have some consistence with observations of agricultural crops, as pathogens within *Cercospora* have been found on soy (Fabaceae) [59]. However, pathogens within *Monographella* have most notably affected agricultural grain (Poaceae) crops, such as rice and corn [60]. Further work is necessary to assess whether information on pathogen specificity on crops is generalizable to other plants.

### Microbiome dynamics can mediate plant biodiversity impacts on function

Our observation of shifts in pathogen composition with plant density (Table 2) is consistent with evidence that pathogen dilution mediates productivity benefits from increased plant species richness [11, 27, 28, 61, 62]. Lower densities of individual host taxa with increasing plant richness could lead to dominance of generalist pathogens [63] or reduced detection of lowered density of specialist pathogens in diverse mixtures, and therefore lower diversity of pathogens at high plant diversity. This pattern was also observed in soil oomycetes (Fig. 3E) and root oomycetes in under-dispersed Poaceae plots. However, dilution of root oomycetes was strongest in mixtures and Fabaceae plots, which may indicate host-specificity of oomycete pathogens on Fabaceae plants, but not in Poaceae or Asteraceae (Fig. 3F). Alternatively, with high host richness, specialists on different taxa could be observed in diverse mixtures with highly sensitive detection environmental sequencing, therefore yielding greater pathogen richness in plots with high richness, as observed in fungal pathogens (Fig. 3C, D). Hence it is possible that both patterns are potentially consistent with dilution of specialized pathogens. At present, we are unable to decipher whether the divergent patterns of oomycete and fungal pathogens results from differing competitive abilities of generalist and specialist pathogens or different sensitivities of environmental sequencing methods.

Other microbial components, such as mutualist AMF, have been found to contribute to plant productivity responses to diversity as well [25, 27]. We found root AMF to marginally decrease in diversity with increased plant species richness (Fig. 3J) and AMF composition was not sensitive to planting design within the time-frame of data collection. The weaker effects we detected indicate that AMF community response to planting design is delayed relative to compared with pathogens and bacteria. Since plant hosts can preferentially allocate resources to symbioses with particular AMF species [64, 65] and AMF species can differentially impact growth of plant taxa [8, 66], increased plant host diversity may dilute positive plant-AMF feedbacks, potentially leading to reduced benefits to preferred plant hosts and reduced productivity with increasing diversity. Our findings provide some insight in that, within a very short time period, pathogens respond more rapidly than AM fungi. How the relative rates of pathogen and mutualist community dynamics might impact plant productivity responses to plant diversity over time needs further investigation.

### Differentiation of soil versus root microbial compartments

We generally observed stronger impacts of plant composition on microbial diversity and composition in roots than soil. This is consistent with strong filters to colonization of roots driving microbe specialization, as might be expected from plant species differing in their signaling for mutualists and immune response to pathogens. Bacteria, while showing weak correlations between soil and root compartments in the co-occurrence analysis (Fig. 2), also showed the most dramatic difference with significant beta dispersion in community composition in response to plant family composition (Fig. 4B). This strong difference in beta dispersion is consistent with a strongly host specific filter to root colonization by bacteria and with the great bacterial diversity in soil being unresponsive to our planting design. In addition, non-pathogenic fungi showed a similar filter to root colonization as did bacteria, though the response is less strong (Table 2). It is possible this difference could be due to portions of the DNA extracted from soil being from non-active cells [67], in which case amplified inactive DNA could have diluted effects of microbial community responses to our experimental design.

Counter to other groups, fungal pathogens had a stronger response in the soil than in roots. Oomycetes also had this trend, although the responses were less dramatic. These responses are consistent with roots colonized by pathogens being turned over quickly [68], thereby releasing the pathogens into the soil. This suggests that fungal pathogens have a relatively faster response to plant community composition than other microbial groups, perhaps because pathogens that have associated with host roots already began moving outwards in the soil to potential new hosts. In plots of low plant species richness and plots with species from only one plant family, the pathogen turnover in the soil is more likely to lead to attack of a nearby susceptible host [21]. This could magnify the growth rates of specialist pathogens, potentially contributing to the significant variation in soil fungal pathogens explained by plant family composition.

### Evidence for home-field advantage for decomposer microbes

We observed strong responses of decomposers (fungal saprotrophs) and potential decomposers (bacteria – unable to match to function) to our planting design, which may indicate proliferation of decomposer microbes that specialize on plant litter types. Soil bacteria (Fig. 4C, D) and root fungal saprobe (Fig. 4E, F) composition differed significantly between plant family treatments, particularly in high diversity plots (Table 2), while root bacteria also had a marginal response (Supplementary Appendix Fig. 3). For all interaction responses, composition of microbial groups in mixtures were central to under-dispersed plant family treatments. Soil bacteria composition becomes more different between composite and legume plots as plant species richness increases (Fig. 4C, D). Similarly, root saprotroph composition becomes more different between grass and composite plots as plant species richness increases (Fig. 4E, F). While we are unable to match bacteria to functional traits at this time, many plant-associated bacteria are decomposers, in addition to some plant mutualists and pathogens [69]. Thus, it is likely that we detect microbial decomposer host-specificity, potentially contributing to the Home-Field Advantage effect [70]. The phylogenetic pattern of differentiation is also consistent with a phylogenetic structure to decomposer home field advantage, as observed in bark [17]. While we do not include plant decomposition rates in congruence with microbial sequencing data, we plan to investigate this relationship in future years of the study.

## Concluding remarks

Just four months after planting this experiment, we observed significant differences in microbial diversity and composition to manipulations of plant community composition, with the strongest observed response in root bacteria and soil fungal pathogens. Microbial pathogens, bacteria and fungal saprotroph communities differentiated between plant family composition, supporting host specificity of pathogens and mutualists. Through both negative plant-soil feedbacks driven by pathogens and positive plant-soil feedbacks driven by host-preferred mutualists, a host plant’s microbiome can mediate plant productivity changes with plant diversity [27, 28, 71]. Future studies need to test the potential for rapid divergence to contribute to plant species coexistence and mediate ecosystem responses to plant diversity.

## Supplementary information

Appendix

## Data Availability

The raw sequencing reads data are uploaded to the NCBI Sequence Read Archive, BIOPROJECT #PRJNA863284. Additional meta data (i.e. planting design) are available from the corresponding author upon reasonable request.
