# Supplementary material for: Rapid differentiation of soil and root microbiomes in response to plant composition and biodiversity in the field
Source: ISME Commun. 2023 Apr 19;3:31. doi: 10.1038/s43705-023-00237-5 (PMC10115818; doi:10.1038/s43705-023-00237-5)
Supplement: Supplementary file 1 — Appendix [file 43705_2023_237_MOESM1_ESM.docx]

*Appendix*

*Appendix Table 1* - Plant species used; 6 per family.

| **Poaceae** | | **Fabaceae** | | **Asteraceae** | |
| --- | --- | --- | --- | --- | --- |
| *Schizachrium scoparium* | SCHSCO | *Amorpha canescens* | AMOCAN | *Liatris pycnostachya* | LIAPYC |
| *Andropogon gerardii* | ANDGER | *Dalea candida* | DALCAN | *Coreopsis tinctoria* | CORTIN |
| *Koeleria macrantha* | KOEMAC | *Dalea purpureum* | DALPUR | *Echinacea pallida* | ECHPAL |
| *Elymus canadense* | ELYCAN | *Desmanthus illinoensis* | DESILL | *Eupatorium altissimum* | EUPALT |
| *Bouteloua gracilis* | BOUGRA | *Desmodium canadense* | DESCAN | *Silphium integrifolium* | SILINT |
| *Panicum virgatum* | PANVIR | *Chamaecrista fasticulata* | CHAFAS | *Helianthus mollis* | HELMOL |


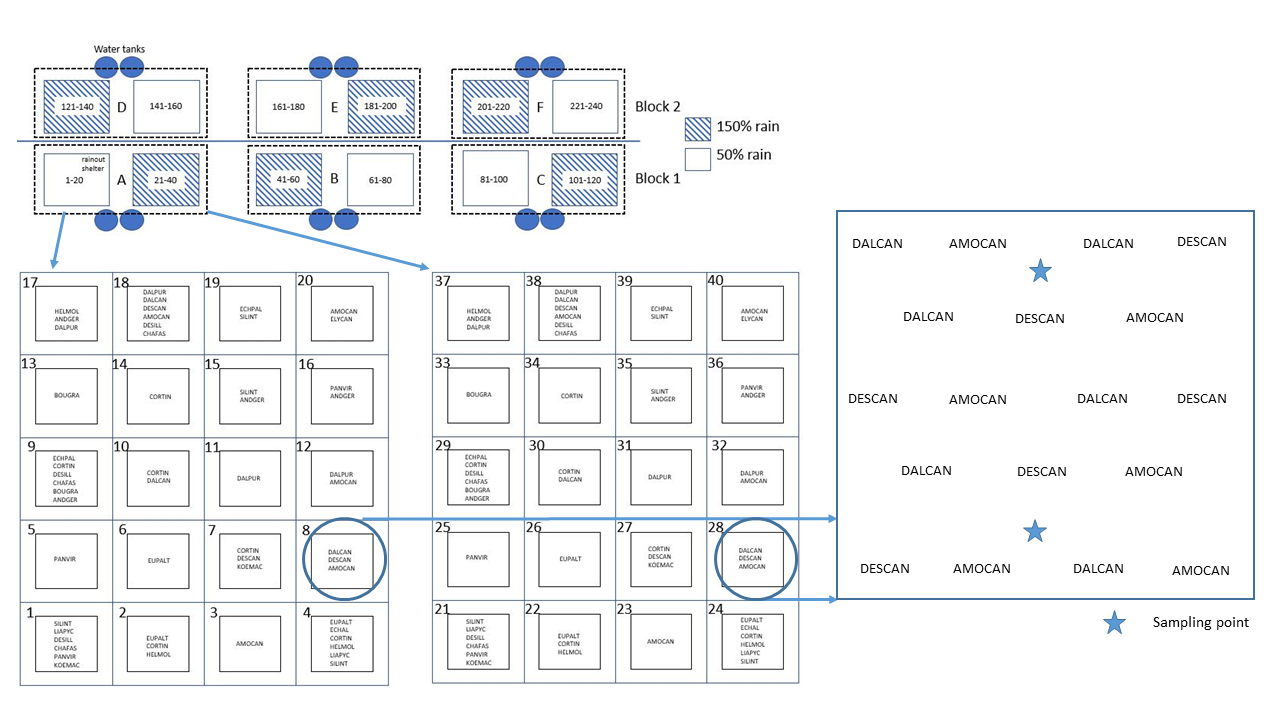


*Appendix Fig. 1* – Experimental plot layout, with Blocks 1 & 2 separated by a dirt road large enough for a small tractor. Subblocks (A-F) have replicate plant design (as seen in zoom of plots 1-20 and 21-40). Each paired subblock design received rainfall manipulations (50% or 150% ambient rainfall) in the years prior to this data collection. This large-scale biodiversity experiment is a multi-year effort beginning in 2018, with many collaborators and subsequent research objectives. The zoomed-in image on the right shows soil sampling points for each plot, taken in 2018, with arrows denoting the pooling of replicate plots. As explained in the methods, we pooled between replicate plots since the rainfall treatment was not implemented yet (first implementation was in 2019 growing season).

*
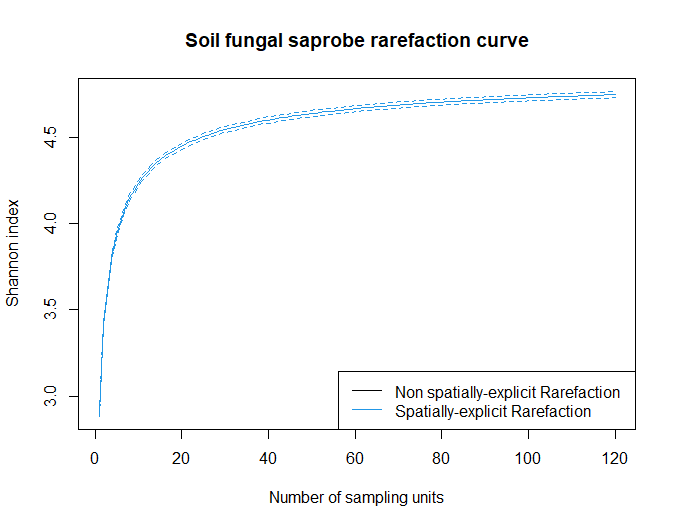

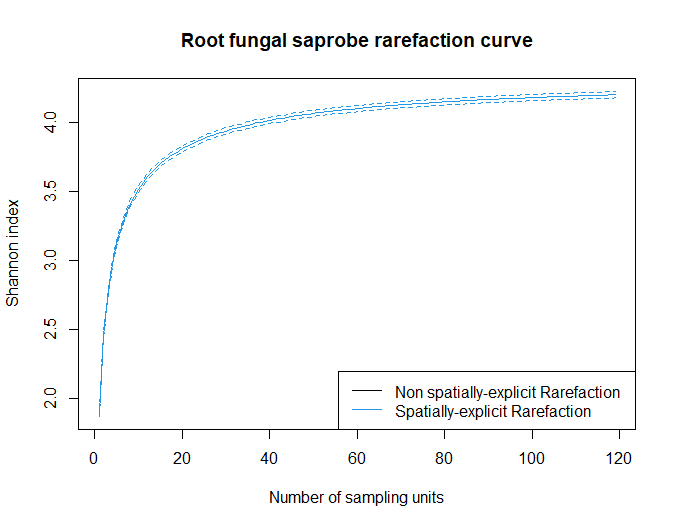
*

*
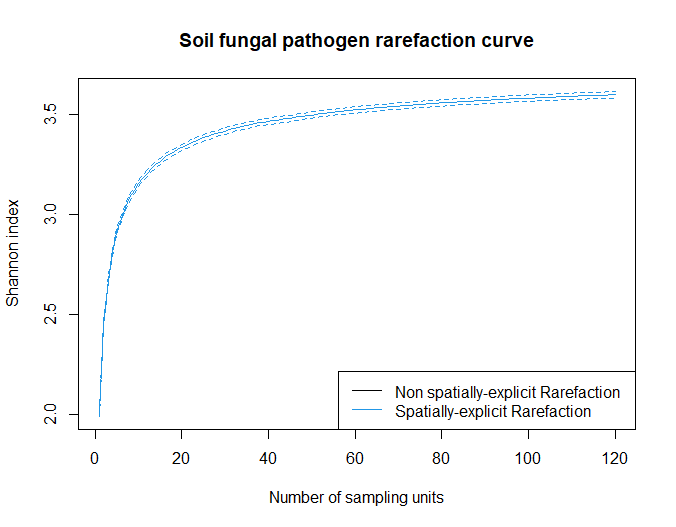

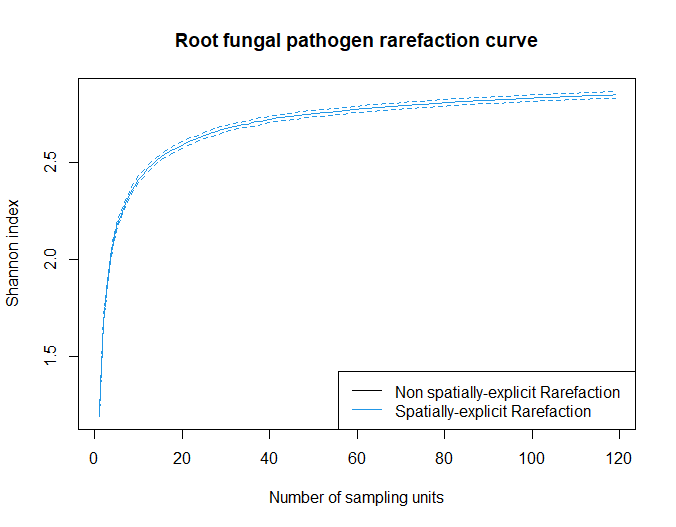
*

*
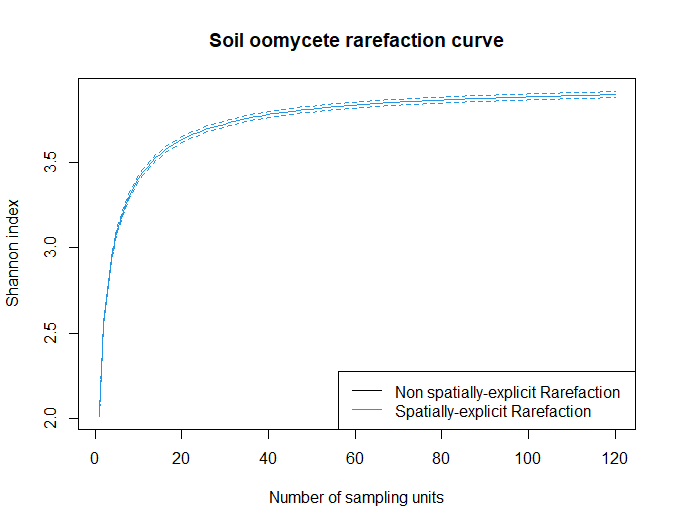

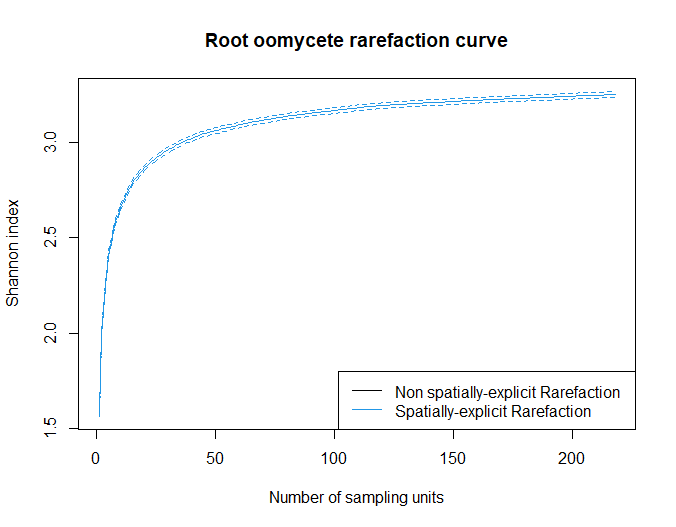
*

*
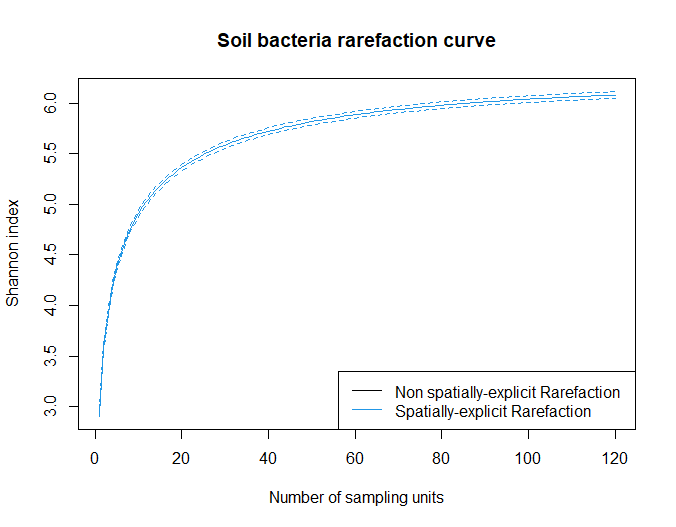

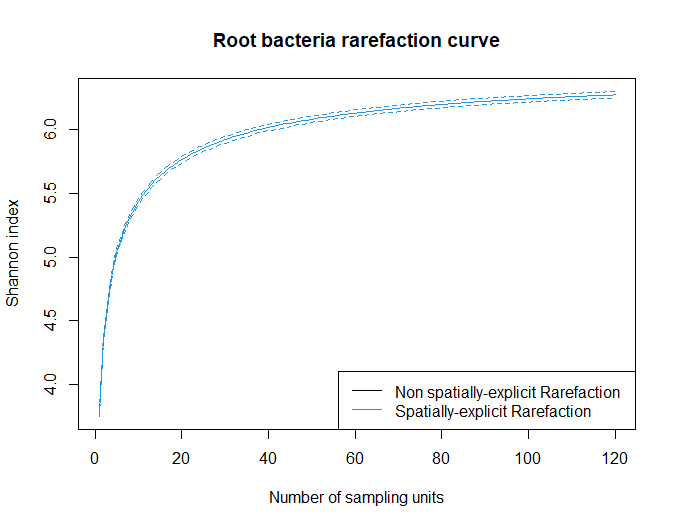
*

*
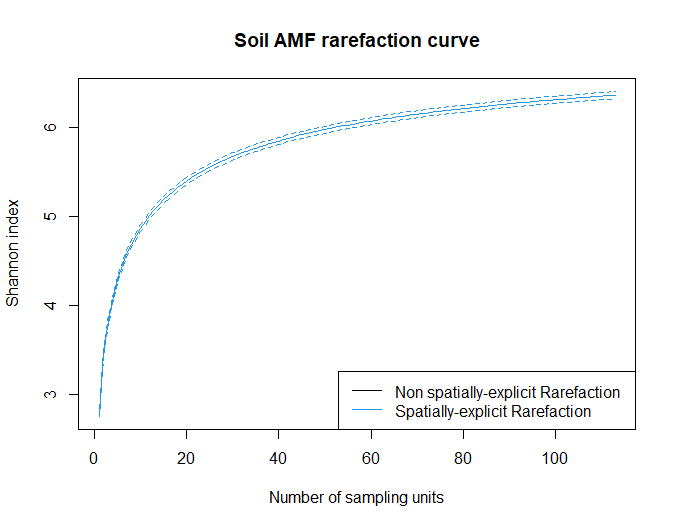

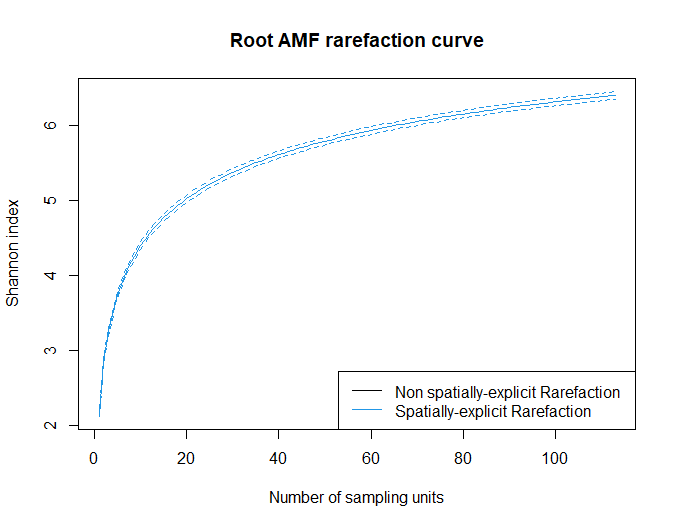
*

*Appendix Fig. 2 -- Rarefaction curves for each sequenced group using adiv package in R. Curves show alpha diversity permutations using raw OTU tables.*

*Appendix Table 2* – Principal coordinate axes for each microbial group that had a significant response to plant family composition or plant species richness in the permanova were then run in linear models with the same model structure to identify axes corresponding to the significant effects in the permanova; paired with Fig. 4. We report significant (p<0.05) as well as marginal effects (p<0.1) from the first 10 principal coordinate axes.

| Soil fungal pathogens |  | PCo 5 | | PCo 6 | | |  |  |  |  |  |  |  |  |  |  |  |  |
| --- | --- | --- | --- | --- | --- | --- | --- | --- | --- | --- | --- | --- | --- | --- | --- | --- | --- | --- |
|  | Df | F-value | Pr(>F) | F-value | Pr(>F) | |  |  |  |  |  |  |  |  |  |  |  |  |
| Plant Sp Richness | 1 | 3.939 | 0.05 | 5.839 | 0.02 | |  |  |  |  |  |  |  |  |  |  |  |  |
| Residuals | 93 |  |  |  |  | |  |  |  |  |  |  |  |  |  |  |  |  |
|  |  |  |  |  |  | |  |  |  |  |  |  |  |  |  |  |  |  |
| Soil bacteria |  | PCo 1 | | PCo 6 | | |  |  |  |  |  |  |  |  |  |  |  |  |
|  | Df | F-value | Pr(>F) | F-value | Pr(>F) | |  |  |  |  |  |  |  |  |  |  |  |  |
| PhyloFam*SpRich | 3 | 2.234 | 0.09 | 2.385 | 0.07 | |  |  |  |  |  |  |  |  |  |  |  |  |
| Residuals | 93 |  |  |  |  | |  |  |  |  |  |  |  |  |  |  |  |  |
|  |  |  |  |  |  | |  | |  |  | |  | | |  |  |  |  |
| Root fungal saprobes |  | PCo 1 | | PCo 3 | | | PCo 7 | |  |  |  |  |  |  |  |  |  |  |
|  | Df | F-value | Pr(>F) | F-value | | Pr(>F) | F-value | Pr(>F) |  |  |  |  |  |  |  |  |  |  |
| Plant Sp Richness | 1 |  |  | 4.29 | | 0.04 |  |  |  |  |  |  |  |  |  |  |  |  |
| PhyloFam*SpRich | 3 | 2.38 | 0.07 |  | |  | 2.24 | 0.09 |  |  |  |  |  |  |  |  |  |  |
| Residuals | 85 |  |  |  | |  |  |  |  |  |  |  |  |  |  |  |  |  |
|  |  |  |  |  |  | |  | |  |  | |  | | |  |  |  |  |
| Root oomycetes |  | PCo 6 | |  |  |  |  |  |  |  |  |  |  |  |  |  |  |  |
|  | Df | F-value | Pr(>F) |  |  |  |  |  |  |  |  |  |  |  |  |  |  |  |
| PhyloFam | 3 | 3.584 | 0.02 |  |  |  |  |  |  |  |  |  |  |  |  |  |  |  |
| Residuals | 93 |  |  |  |  |  |  |  |  |  |  |  |  |  |  |  |  |  |
|  |  |  |  |  |  | |  | |  |  |  | |  |  |  |  |  |  |
| Root bacteria |  | PCo 1 | | PCo 2 | | | PCo 5 | |  |  |  |  |  |  |  |  |  |  |
|  | Df | F-value | Pr(>F) | F-value | Pr(>F) | | F-value |  |  |  |  |  |  |  |  |  |  |  |
| Plant Sp Richness | 1 |  |  | 4.98 | 0.03 | | 4.96 | 0.03 |  |  |  |  |  |  |  |  |  |  |
| PhyloFam | 3 | 17.4 | 4.8E-9 | 54.25 | 2.2E-16 | |  |  |  |  |  |  |  |  |  |  |  |  |
| PhyloFam*SpRich | 3 | 2.40 | 0.07 | 2.26 | 0.09 | |  |  |  |  |  |  |  |  |  |  |  |  |
| Residuals | 93 |  |  |  |  | |  |  |  |  |  |  |  |  |  |  |  |  |


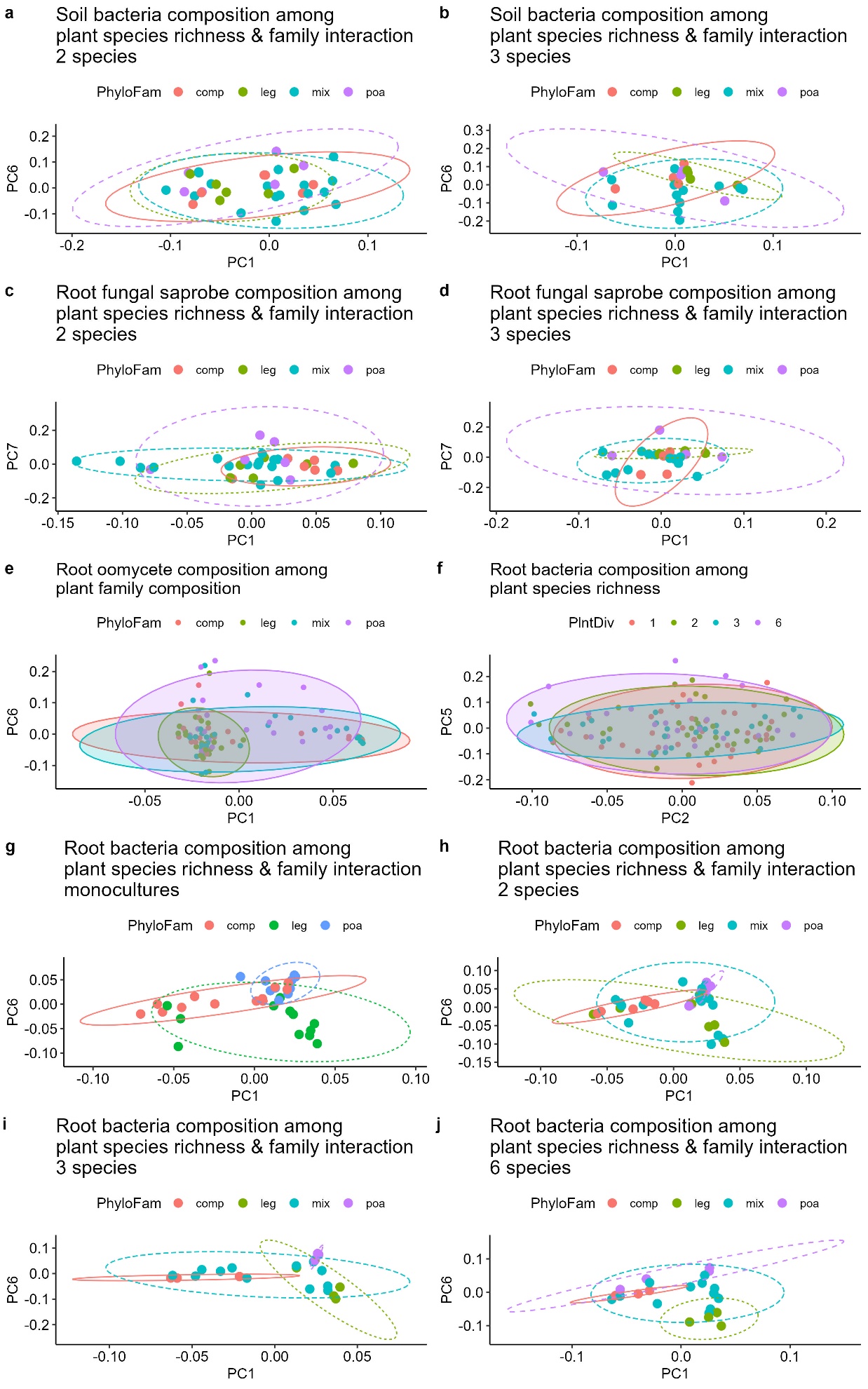


*Appendix Fig. 3* – Extended panels of Fig. 4, pcoa axes that are driving significant responses in the permanova, see appendix table 3 for linear response of each axis and table 2 of manuscript for permanova response. a-d, g-j show interactions not depicted in Fig. 4: soil bacteria interaction for 2 species (a) and 3 species (b) plots, root fungal saprobe interactions for 2 (c) and 3 (d) species plots, and root bacteria marginal interaction response for all plant species richness treatments (g-j). Root oomycete marginal response to plant family composition (e) and root bacteria marginal response to plant species richness treatment (f) are also included here as visual aid.

*Appendix Table 3* – Anova output for Soil fungal pathogen genera relative abundance that differed significantly between phylogenetic groups (see PhyloFam response).

|  |  | Monographella | | Cercospora | | Erysiphe | | Stagonospora | |
| --- | --- | --- | --- | --- | --- | --- | --- | --- | --- |
|  | **Df** | **F value** | **Pr(>F)** | **F value** | **Pr(>F)** | **F value** | **Pr(>F)** | **F value** | **Pr(>F)** |
| Block | 1 | 0.727 | 0.396 | 4.161 | 0.044 | 0.353 | 0.554 | 2.094 | 0.152 |
| PhyloFam | 3 | 5.093 | **0.003** | 3.200 | **0.027** | 3.942 | **0.011** | 2.468 | **0.067** |
| PlntDiv | 3 | 0.496 | 0.686 | 4.316 | **0.007** | 0.570 | 0.636 | 1.164 | 0.328 |
| SCHSCO | 1 | 0.107 | 0.744 | 0.039 | 0.844 | 0.548 | 0.461 | 0.448 | 0.505 |
| ANDGER | 1 | 0.050 | 0.823 | 0.501 | 0.481 | 0.152 | 0.698 | 6.403 | **0.013** |
| KOEMAC | 1 | 0.124 | 0.726 | 0.115 | 0.736 | 0.283 | 0.596 | 7.287 | **0.008** |
| ELYCAN | 1 | 0.691 | 0.408 | 0.686 | 0.410 | 0.815 | 0.369 | 0.907 | 0.344 |
| BOUGRA | 1 | 0.003 | 0.956 | 0.078 | 0.781 | 0.522 | 0.472 | 0.007 | 0.934 |
| PANVIR | 1 | 1.713 | 0.194 | 0.469 | 0.495 | 1.196 | 0.277 | 2.474 | 0.119 |
| AMOCAN | 1 | 2.597 | 0.111 | 0.065 | 0.800 | 1.149 | 0.287 | 0.162 | 0.688 |
| DALCAN | 1 | 7.061 | **0.009** | 0.012 | 0.914 | 4.708 | **0.033** | 1.909 | 0.171 |
| DALPUR | 1 | 0.361 | 0.550 | 0.249 | 0.619 | 5.041 | **0.027** | 0.747 | 0.390 |
| DESILL | 1 | 4.710 | **0.033** | 0.024 | 0.878 | 32.515 | **0.000** | 0.404 | 0.527 |
| DESCAN | 1 | 10.646 | **0.002** | 0.339 | 0.562 | 4.268 | **0.042** | 0.333 | 0.565 |
| CHAFAS | 1 | 4.054 | **0.047** | 0.913 | 0.342 | 0.154 | 0.695 | 0.546 | 0.462 |
| LIAPYC | 1 | 0.648 | 0.423 | 0.005 | 0.947 | 0.108 | 0.744 | 0.070 | 0.792 |
| CORTIN | 1 | 0.133 | 0.716 | 0.808 | 0.371 | 0.142 | 0.707 | 6.543 | **0.012** |
| ECHPAL | 1 | 0.076 | 0.783 | 0.140 | 0.709 | 1.038 | 0.311 | 0.000 | 0.994 |
| EUPALT | 1 | 0.041 | 0.841 | 0.266 | 0.607 | 0.118 | 0.732 | 0.186 | 0.667 |
| SILINT | 1 | 0.099 | 0.754 | 0.003 | 0.955 | 0.001 | 0.978 | 0.003 | 0.960 |
| HELMOL | 1 | 0.094 | 0.760 | 0.051 | 0.822 | 0.017 | 0.896 | 0.329 | 0.568 |
| Phylo:Div | 8 | 2.538 | **0.016** | 1.695 | 0.111 | 0.797 | 0.606 | 0.827 | 0.581 |


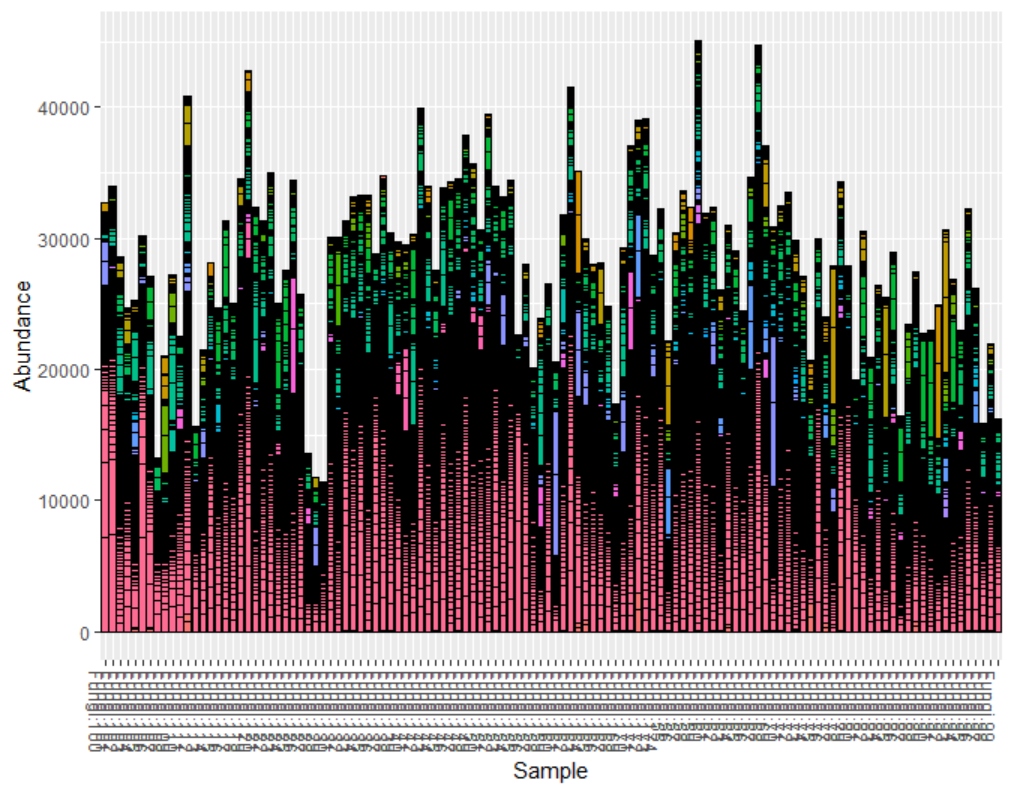


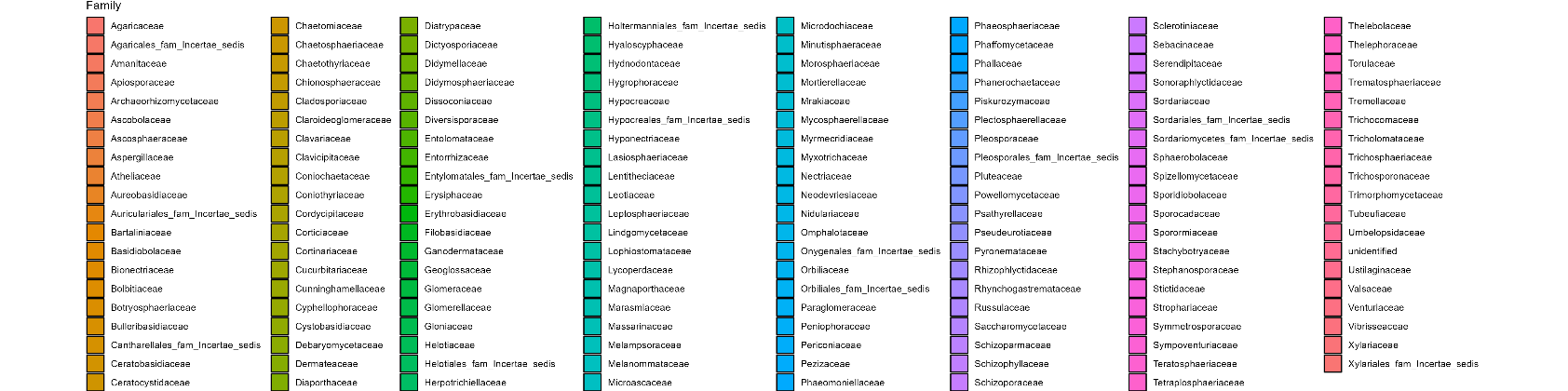


*Appendix Fig. 4 – Soil fungal OTU abundance by fungal family per plot on x-axis. These are filtered OTUs, before center-log ratio transformations.*
